# Evaluation of a Concrete Slab Track with Debonding at the Interface between Track Concrete Layer and Hydraulically Stabilized Base Course Using Multi-Channel Impact-Echo Testing

**DOI:** 10.3390/s21217091

**Published:** 2021-10-26

**Authors:** Jin-Wook Lee, Sung-Jin Lee, Seong-Hoon Kee

**Affiliations:** 1 Advanced Railroad Civil Engineering Division, Korea Railroad Research Institute, 176 Cheoldobangmulgwan-ro, Uiwang-si 16105, Gyeonggi-do, Korea; jinugi@krri.re.kr; 2 Department of ICT Integrated Ocean Smart Cities Engineering, Dong-A University, 37 Nakdong-daero 550, Beon-gil, Saha-gu, Busan-si 69315, Korea

**Keywords:** concrete slab track, debonding defects, multi-channel IE testing, data reduction, non-destructive evaluation

## Abstract

Multi-channel Impact-echo (IE) testing was used to evaluate debonding defects at the interface between track concrete layer, TCL, and hydraulically stabilized base course, HSB, in a real scale mockup model of concrete slab tracks for Korea high-speed railway (KHSR) system. The mockup model includes three debonding defects that were fabricated by inserting three 400 mm by 400 mm (length and width) thin plastic foam boards with three different thicknesses of 5 mm, 10 mm, and 15 mm, before casting concrete in TCL. Multi-channel IE signals obtained over solid concrete and debonding defects were reduced to three critical IE testing parameters (the velocity of concrete, peak frequency, and *Q* factor). Bilinear classification models were used to evaluate the individual and a combination of the characteristic parameters. It was demonstrated that the best evaluation performance was obtained by using average peak frequency or the combination of average peak frequency and average *Q* factor, obtained by eight accelerometers in the multi-channel IE device. The results and discussion in this study would improve the understanding of characteristics of multiple IE testing parameters in concrete slab tracks and provide a fundamental basis to develop an effective prediction model of non-destructive evaluation for debonding defects at the interface between TCL and HSB in concrete slab tracks.

## 1. Introduction 

Concrete slab tracks (or ballastless tracks) are widely used in the high-speed railway systems in Germany, the Netherlands, China, Japan, and Korea [1,2]. The ballastless track is superior to the conventional ballast track due to its higher track stability, more favorable riding comports, longer service life, and especially lower maintenance cost [2,3,4,5,6]. There are two types of ballastless concrete tracks developed for the high-speed railway system in Korea: (1) continuous concrete slab tracks fabricated using cast-in-place concrete and (2) discrete concrete slab tracks made of precast concrete (PC) [7,8]. The continuous concrete slab tracks have been increasingly used in the Korea high-speed railway (KHSR) system after first being adopted to the second section of the Gyeongbu line (Daegu~Busan) and the Honam line (Osong~Gwangju Songjeong) [9]. The PC tracks have been developed and first constructed in a test section of the Honam line. However, it has not been adopted to the field practice yet [10]. 

**Figure 1:**
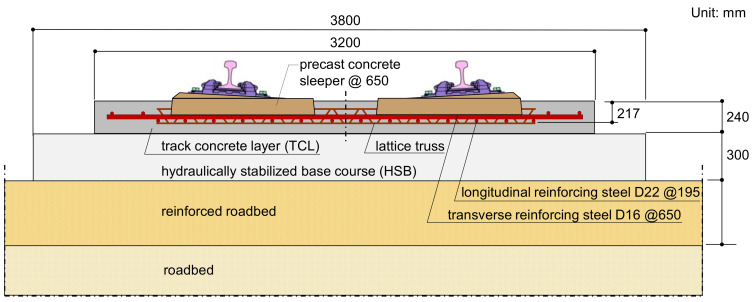
Standard section of the continuous concrete slab track in high-speed railway system in Korea (Korea Continuous track, KCT, II system), developed based on the German Rheda 2000 system. The KCT II system was adopted to the 2nd section of the Gyeongbu line (Daegu~Busan).

The continuous concrete slab tracks in Korea were developed based on the German Rheda 2000 system [9,11,12] (see Figure 1). The track concrete layer (TCL) is a longitudinally continuous reinforced concrete slab without construction joints at the section of earthworks and tunnels but is discontinuous on bridges. Two PC railway sleepers, connected by lattice trusses, are embedded into the cast-in-place concrete slab in TCL. Steel rails with 60 kg/m are attached to the railway sleeper using a high elastic fastening system. The TCL is supported by a hydraulically stabilized base course (HSB) that is constructed using cast-in-place concrete without steel reinforcements. The two concrete layers (i.e., TCL and HSB) in the concrete railway track are connected by an interface adhesion to resist internal stresses caused by cyclic train dynamic loads and environmental effects. In addition, the double-layered slab is continuously laid on the subgrade, which imposes a fixed boundary condition at the interface between the bottom of the HSB and the top surface of the subgrade. 

**Figure 2:**
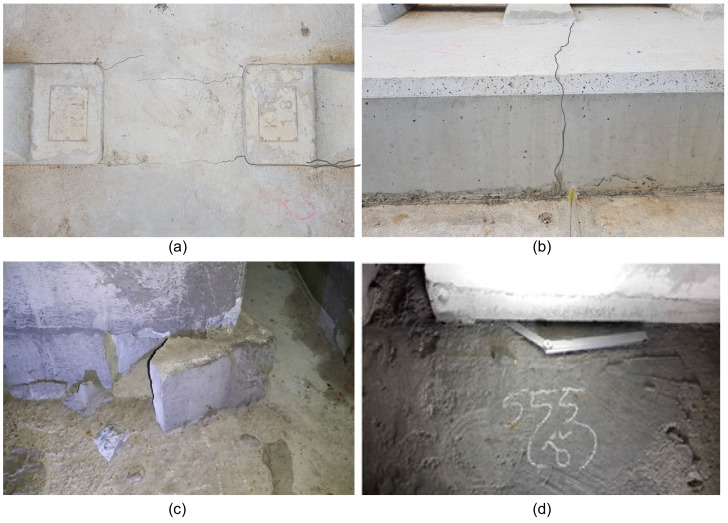
Typical cracks and damages in concrete slab tracks in Korea: (**a**,**b**) surface-breaking cracks in the continuous concrete slab track in a high-speed railway system in Korea and (**c**,**d**) damages in precast concrete railway tracks in an urban subway system in Korea.

Various defects (surface-breaking cracks and internal damages) have been observed on the actual concrete slab tracks in the KHSR system [5]. Typical types of cracks in TCL are shown in Figure 2. The most frequently observed pattern is the presence of transverse cracks on the surface of TCL that was generally initiated at the interface between PC sleepers and TCL concrete, particularly at the section of earthworks. Furthermore, the separation of PC sleepers and TCL was noticed in some survey regions. The presence of surface-breaking cracks does not necessarily result in the degradation of the structural performance of railway tracks. However, it can induce major damage in concrete, such as debonding at the interface between TCL and HSB, which is a major topic in this study. Recently, the authors obtained field test results indicating that some of the surface-breaking cracks could induce horizontal cracks in concrete at the interface between TCL and HSB. The interface damage could reduce the structural performance of concrete slabs (e.g., stiffness, strength, and stability of the entire system) and cause local buckling or longitudinal instability of the track system [2,4,5,13,14,15]. The interface defects in concrete have been more frequently observed in PC concrete tracks. From the standpoint of the railway infrastructure management agency, it is of importance to evaluate the condition of critical elements in concrete tracks, and if necessary, to make an appropriate decision for the maintenance of concrete railway tracks. As such, it will assure safety and favorable riding comports for public users. 

The overall condition of concrete slab tracks is estimated through visual inspection in a periodic inspection plan in Korea. Visual inspection is primarily used to investigate any surface evidence associated with damages in concrete slabs (e.g., cracks, popouts, scaling, spalling, efflorescence, and discoloration due to steel corrosion). However, the information gathered through visual inspection is only limited to the surface, thus, it is challenging to evaluate sub-surface damages (e.g., horizontal cracks such as delamination and debonding defects, honeycombs, etc.) in concrete. In order to collect additional information on the sub-surface defects, field engineers usually tap the surface of concrete using a small hammer. The test region over sub-surface damages can be identified by hollow (or drummy) sounding [16]. The hammer tapping (or sounding) method has been demonstrated to be effective to detect and evaluate the relatively shallow horizontal cracks in concrete structures. Theoretically, the drummy sounding is associated with the flexural vibration of concrete over its defects. The excitability of flexural vibration mode is dependent on mechanical properties (elastic modulus, mass density, and Poisson’s ratio) and the dimensional size (depth and width) of concrete over defects [17,18]. Concrete with a greater width to depth ratio results in greater excitability of flexural vibration mode. It was noticed from the laboratory and field tests that the hammer tapping method is not sufficient to generate discernible hollow sounding when applied to relatively deep debonding defects in concrete slabs [17,18,19,20,21,22,23]. 

The impact-echo (IE) test has been demonstrated to be an effective evaluation of defects in concrete plates in buildings and civil infrastructure systems [24,25]. The IE test measures the dynamic responses of concrete by applying an impact point loading on the surface of concrete. Unlike the hammer tapping method, the IE test uses vibration or an acoustic sensor (accelerometer, displacement sensor, and microphone) to measure the dynamic response of concrete in structures. As such, it enables the measurement of even weak signals that human ears cannot sense. There are many prior research works that successfully demonstrated the capability of IE testing for evaluating the horizontal cracks in concrete in laboratory and field practice. According to the conventional IE theory, there are two sets of resonance modes associated with Lamb waves in slabs to evaluate horizontal defects in concrete: the thickness stretch mode and flexural vibration mode, which will be described in the background section of this study. However, it has been argued that the IE signals measured in actual concrete structures contain various unknown signals, which could not be explained by the conventional IE theory. There are several sources of unknown signals in the field practice. First, actual concrete structures include various types of embedded items during construction (e.g., reinforcing steel, spacers, utility pipes, and other concrete elements) that could generate various resonance waves in concrete [26]. Second, concrete elements in actual structures have limited dimensional sizes from which multiple reflection signals are set up [27]. Third, concrete is a heterogenous material composed of different inorganic materials such as cement, sand, gravel, and other chemical components. Some reflected waves could be generated by the interface between different materials. Furthermore, the property of concrete is dependent on the construction methods (packing, curing, location of elements, etc.) and environmental conditions (humidity, temperature, water saturation, etc.). Therefore, it is necessary to understand the characteristics of IE data from solid and defected concrete in actual structures for the successful use of IE testing for non-destructive evaluation methods in the field practice. Unfortunately, it is difficult to find relevant sources in the literature that describe the IE testing data on the actual concrete slab tracks. 

The primary objectives of this research are to evaluate the multi-channel IE testing parameters measured on a real sale mockup model of concrete slab tracks for the Korea high-speed railway (KHSR) system and to propose a bilinear classification model for non-destructive evaluation of debonding defects in concrete slab tracks by multi-channel IE testing. For these purposes, three main tasks were performed in this study. First, multi-channel IE testing was performed on a real scale mockup model of concrete slab tracks that includes three debonding defects at the interface between TCL and HSB. Second, multi-channel IE signals obtained over solid concrete and debonding defects were reduced to three critical IE testing parameters (i.e., the velocity of concrete, peak frequency, and *Q*-factor). Third, bilinear classification models were used to evaluate the individual and a combination of the characteristic parameters. As will be discussed, the use of multi-channel IE parameters results in a more accurate prediction model that evaluates the debonding defects in the concrete slab tracks. 

## 2. Background

**Figure 3:**
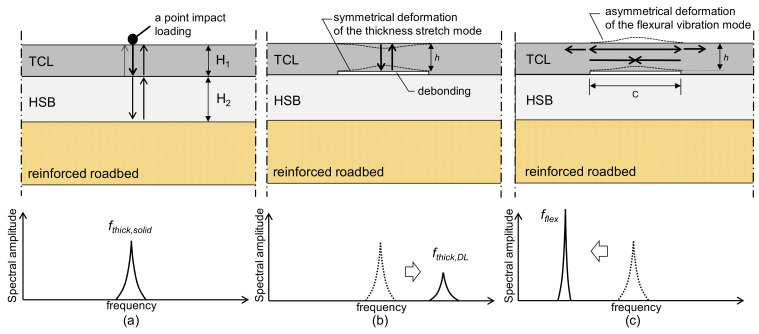
Illustration of Impact-echo (IE) test on a concrete slab track with double-layer concrete slabs (track concrete layer, TCL, and hydraulically stabilized based course, HSB): (**a**) dominated by the thickness stretch mode over solid concrete with the thicknesses of TCL and HSB, *H_1_* and *H_2_*, (**b**) dominated by the thickness stretch mode over a debonding defect of the depth of *h*, and (**c**) dominated by the flexural vibration mode over a debonding defect of the width of *c*.

The IE test is an NDE method that measures the dynamic response of concrete induced by an impact point loading. The mechanical impact applied on the surface of the concrete generates body waves and surface-guided waves that propagate in the concrete plate. Figure 3 illustrates possible dominant waves generated by IE testing on the concrete slab track with a double-layer concrete slab (TCL and HSB). IE test over solid concrete and over debonding at the interface between TCL and HSB are shown in Figure 3a–c, respectively. 

Two sets of resonance modes associated with Lamb waves in slabs are generally used to detect and characterize horizontal defects in concrete: the thickness stretch mode and flexural vibration mode. The frequency analysis of the two resonance modes provides useful information on the presence and dimensions of horizontal defects (areal extensions and depths) in concrete. First, the thickness stretch mode dominates the spectral response of a plate that does not contain any near-surface defects (see Figure 3a). Thickness stretch mode results from multiple reflections of bulk waves (P- and S-waves) between the top and bottom surfaces of a solid plate. In solid concrete, a thickness stretch mode is induced by resonance waves that are generated by multiple reflections of bulk waves at the bottom surface of HSB and the top surface of TCL (Figure 3a). For the free (or single-layered) plate, the thickness stretch mode frequency is simply represented in terms of P-wave velocity (*C_p_*) and the thickness (*H*) of the plate as follows,

(1) $$f_{thick,single}=\;\frac{\beta C_{p}}{2H}$$

where β is a correction factor that depends on Poisson’s ratio of concrete and is in a range of 0.945 to 0.967 for concrete with Poisson’s ratio from 0.16 to 0.25 [28]. For the double-layered plate, the thickness mode frequency is represented in terms of P-wave velocities and thicknesses of the two slabs as follows,

(2) $$f_{thick,double}=\;\frac{C_{p1}C_{p2}}{2\beta_{1}C_{p1}H_{1}+2\beta_{2}C_{p2}H_{2}}$$

where subscripts 1 and 2 indicate the properties of TCL and HSB, respectively. Furthermore, the thickness stretch mode frequency could be shifted in the high-frequency direction if there is a debonding defect in concrete between TCL and HSB (see Figure 3b). The thickness stretch mode is set up by the multiple reflections of the bulk waves between the free surface of concrete and the top surface of debonding defect. Equation (1) is effective for estimating the thickness stretch mode frequency over a debonding defect by replacing *H* with the depth of the debonding defect in concrete, *h*, as follows,

(3) $$f_{thick,DL}=\;\frac{\beta_{1}C_{p1}}{2h}$$

The size of a debonding defect should be large enough compared to the depth of the defect to set up the thickness stretch mode in concrete. There is a shadow zone over a perimeter of the defect that does not produce clear thickness stretch mode. As a rule of thumb, the size of the delamination defect should be about two times greater than the depth of the delamination defect and both sensor and source should be located over the middle of the delamination defect avoiding the shadow zone. The relatively strict criteria pose difficulties on the use of thickness stretch mode to evaluate the horizontal defects in concrete, especially in the field practice. 

In contrast, debonding defects in concrete can be characterized by flexural vibration mode. Flexural vibration modes can be interpreted as non-propagating waves because of multiple reflections of Lamb waves at a debonding defect. The propagating Lamb waves (A0 and S0) over a debonding defect are reflected and transmitted multiple times at the edge of the debonding defects [29]. Unlike the thickness stretch mode, the flexural vibration mode is less sensitive to the location of the impact source: that is, it can be generated by an impact source located over or out of the defect [17,18]. Therefore, a more flexible test plan can be made to detect a delamination defect by the flexural vibration measurements. The resonance frequency of the flexural vibration mode, *f_flex_*, is dependent on the plate geometry (lateral dimensions and depth), shape, and boundary conditions [20]. The analysis of *f_flex_* is effective for evaluating the presence of relatively shallow delamination defects compared to the depth of the defect in concrete. It is also useful to estimate the areal extents of the debonding defects [17,18,19,20,23] by using the semi-analytical equation that relates the fundamental frequency of the flexural vibration mode over delamination defect, *f_flex_*. It can be expressed by mechanical properties of concrete and dimensional sizes of concrete over the square debonding defects (*h* and *h*/*c*) [20] as follows,

(4) $$f_{flex}\;=\varepsilon \beta_{2}\frac{C_{p1}}{h}{\left(\frac{h}{c}\right)}^{2}\;\;\;\;\;\;\;\;\;\;\;\;\;\;\;for\;\;\;\frac{c}{h}\;\geq \;1,$$

where *h* and *c* is the depth and the width of a debonding defect, respectively, and ε is the correction factor for edge effects of the fundamental flexural vibration mode that is written as follows,

(5) $$\varepsilon \;=\;\eta_{1}e^{\eta_{2\left(c/h\right)}}+\eta_{3}e^{\eta_{4\left(c/h\right)}},$$

where *η*_1_, *η*_2_, *η*_3_, and *η*_4_ are constants, whose values are 1.64, 0.0014, −1.812, −0.22, respectively. $\beta_{2}$ is a correction factor that depends on Poisson’s ratio of concrete as follows,

(6) $$\beta_{2}=\frac{\pi }{2}\frac{\sqrt{1-2\upsilon }}{\sqrt{3{\left(1-\upsilon \right)}^{2}}}$$

**Figure 4:**
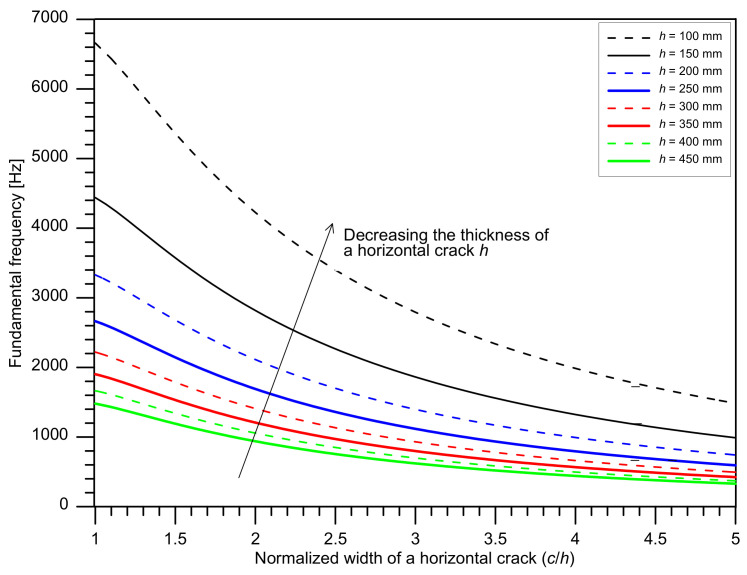
Variation of the fundamental frequency of the flexural virbation mode over a horizontal crack with normalized width (*c*/*h*, the width of a defect, *c*, normalized by the depth of a defect, *h*) and the thickness of a defect, *h*. Figure 4 was drawn based on the materials’ properites with Poisson’s ratio of 0.18 and P-wave velocity of 4000 m/s.

Figure 4 shows the variation of the fundamental frequency of flexural vibration mode *f_flex_* over a debonding defect with normalized width (the width of a defect, *c*, normalized by the depth of a defect, *h*) and thickness of a debonding defect. Figure 4 was drawn based on the material properties with Poisson’s ratio υ of 0.18 and P-wave velocity $C_{p}$ of 4000 m/s. The curve corresponding to the depth of *h* = 250 mm in Figure 4 represents the predicted fundamental frequency of flexural vibration mode over a debonding defect at the interface between TCL and HSB.

## 3. Method

### 3.1. Test Specimen

**Figure 5:**
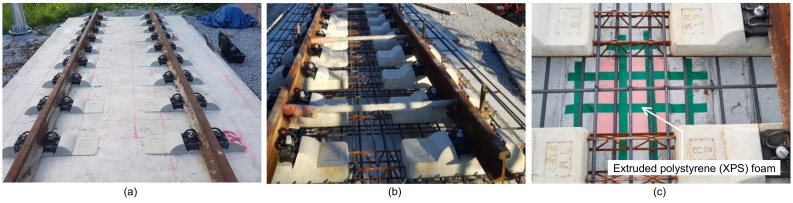
A real scale mockup model of concrete slab tracks in the Korea high-speed railway (KHRS) system used in this study: (**a**) a picture of a part of the concrete slab track specimen, (**b**) embedded items in the track concrete layer, TCL, concrete (precast concrete sleepers, lattice trusses, reinforcing steels and various inserts) and rails, (**c**) an extruded polystyrene (XPS) foam board located on the surface of hydraulically stabilized based course, HSB, to fabricate artificial debonding at the interface between TCL and HSB.

Figure 5a shows a real-scale mockup model of concrete slab tracks for the KHSR system that was fabricated at Dong-Yang University as part of a research project to develop innovative condition assessment and rehabilitation methods for preventive maintenance of concrete slab track systems in Korea. The concrete slab track specimen was designed based on the Rheda 2000 system (see Figure 1). Figure 5b shows embedded items in the TCL concrete slab during the construction of the concrete slab track specimen, which includes PC sleepers, lattice trusses, reinforcing steels, and various inserts. The concrete slab specimen was designed to contain three fabricated debonding defects at the interface between TCL and HSB. Extruded polystyrene (XPS) foam boards with a dimensional size of 400 mm by 400 mm (length and width) and three different thicknesses (5 mm, 10 mm, and 15 mm) were placed on the HSB layer before casing concrete for TCL. Consequently, the depths to the debonding defects were 235 mm, 230 mm, and 225 mm, respectively. Figure 5c shows a picture of the installation of an XPS foam board on the HSB layer in the concrete slab track. 

### 3.2. Multi-Channel IE Data Measurements

**Figure 6:**
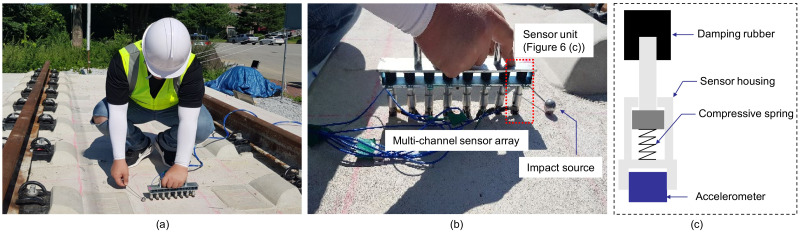
Impact-echo testing on the concrete slab track specimen using a multi-channel IE testing device: (**a**) IE testing on the concrete slab track specimen, (**b**) the multi-channel sensor array used in this study, and (**c**) a sensing unit in the multi-channel sensor array that is composed of an accelerometer, a sensor housing, a compressive spring, and a damping rubber.

A multi-channel IE testing device, developed by a research team at Dong-A University, was used to evaluate the concrete slab track specimen (see Figure 6a). The prototype equipment is composed of a multi-channel sensor array with eight sensing units, signal conditioners, a data acquisition system, and a laptop computer. Specifics of the multi-channel IE device are described in another publication [19].

**Figure 7:**
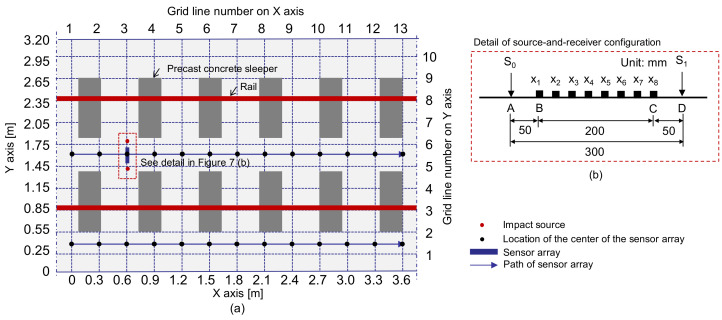
Test plan on the surface of solid concrete: (**a**) scanning procedure for multi-channel Impact-echo test and (**b**) source-and-receiver configuration.

**Figure 8:**
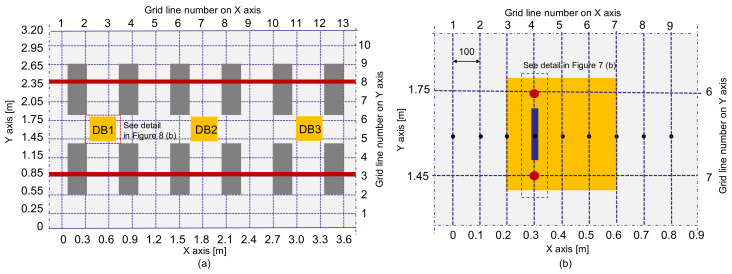
Test plan on the concrete surface over debonding defects: (**a**) location of debonding defects (DB1, DB2, and DB3) and (**b**) test plan over a debonding defect.

The multi-channel IE testing device was used to measure the dynamic responses of four test regions on the concrete slab track specimen. One test region represents the solid concrete (Figure 7), and the other three regions include fabricated debonding defects at the interface between TCL and HSB (Figure 8). For the solid concrete region, IE test was performed along the two horizontal lines defined by Y = 0.4 m and Y = 1.6 m, respectively (Figure 7a); while IE test was performed along the one horizontal line defined by Y = 1.6 m in the three test regions over debonding defects (Figure 8a). Black solid circles in Figure 7a and Figure 8b represent the IE test points in the test regions over solid concrete and debonding defects. The distances between two adjacent testing points in the solid and de-bonded test regions are 300 mm and 100 mm, respectively. For the IE test at each test point, the sensor array was placed at the test point, which was kept perpendicular to the horizontal axis (the path of IE testing) with the middle of the sensor at each test point. A steel ball with a diameter of 20 mm was used to generate incident elastic waves that have enough energy penetrating into relatively deep thicknesses (240 mm for TCL and 300 mm for HSB) of the concrete slab specimen in this study. First, incident elastic waves were generated by an impact loading applied at the location of S0, which is on the left side of the sensor array and was measured by the eight sensors simultaneously. Next, an independent test was repeated by applying an impact loading at S1, which is on the right side of the sensor array. Then, the elastic wave measurement was repeatedly performed along the test line as shown in Figure 7 and Figure 8. The data scans represented a total of 52 and 54 IE tests on the surface of solid and debonding defects regions, respectively.

### 3.3. Typical Signals and IE Data Anlaysis

**Figure 9:**
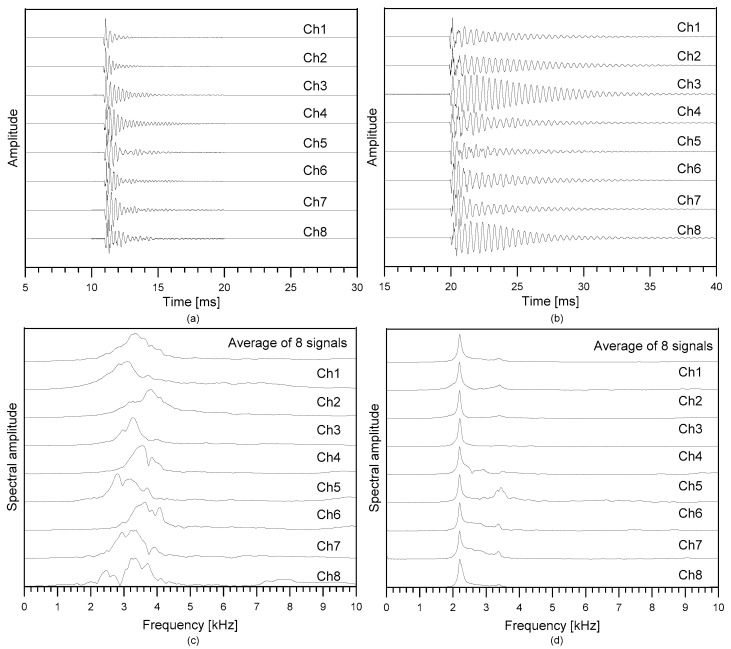
Typical signals measured by the multi-channel IE testing device on concrete over the solid concrete and a debonding defect: (**a**,**b**) typical time signals measured over solid concrete and a debonding defect, and (**c**,**d**) spectral amplitude signals corresponding to the time signals shown in (**a**,**b**), respectively.

Figure 9a,b show typical time signals measured over the solid test region and the debonding defect in test region 3, respectively (see Figure 7 and Figure 8). A total of eight acceleration signals were measured by the multi-channel IE testing device at a single impact loading. The time signals in Figure 9a were measured by locating the middle of the sensor array at *r* = (*x_r_*, *y_r_*) = (1.8 m, 0.40 m) and the impact source at *s* = (*x_s_*, *y_s_*) = (1.8 m, 0.55 m). The time signals shown in Figure 9b were measured by locating the middle of the sensor array at *r* = (*x_r_*, *y_r_*) = (3.15 m, 1.6 m) and *s* = (*x_s_*, *y_s_*) = (3.15m, 1.75 m). 

The spectral amplitude of the dynamic response of concrete was obtained by the Fourier transform of the time signals (see Figure 9c,d). The spectral amplitude exhibits a unique clear peak frequency. The peak frequencies measured on the sound concrete are ranging from 3000 Hz to 3500 Hz, which are close to the thickness stretch mode predicted by Equation (2) (~3300 Hz). Therefore, the peak frequency in this range could be interpreted as the characteristic frequency of the sound concrete region. In addition, there are secondary peaks around 2000 Hz and 4000 Hz, which are not expected by the conventional IE theory. The peak frequency values in this range are likely due to the unknown acoustic reflectors in TCL concrete. In contrast, the spectral response measured on the concrete over a debonding defect was dominated by a clear peak frequency of about 2200 Hz, which shows a good correlation with the predicted flexural vibration mode frequency (~2100 Hz) by Equations (4)–(6). Therefore, the peak frequency could be an effective parameter to classify the solid and debonding regions. 

In addition, it was noticed that spectral amplitude signals corresponding to the flexural vibration mode are even clearer peaks compared to the spectral response from the thickness stretch mode in the sound concrete. In this study the sharpness, of the spectral amplitude was considered as a critical IE testing parameter to evaluate the debonding defects in the concrete slab track specimen. In general, the differences in the sharpness of the special amplitude are caused by different damping properties of vibration signals. The *Q* factor was used as a measure of the damping property of the oscillation signal (or the energy lost in the cycle of oscillation). In this study, the *Q* factor was determined by normalizing a center frequency of a resonance mode (*f_c_*) by its bandwidth (*B*) excited by an impact force on each testing point as follows,

(7) $$Q\;factor=\frac{f_{c}}{B}$$

where *B* is the bandwidth determined from the difference between the upper frequency (*f_H_*) and lower frequency (*f_L_*), in a −3 dB lower power limit. 

A unique benefit of using a multi-channel IE device, compared to a single sensor IE device, is its capability of measuring the velocity of concrete at a single IE testing. The velocity of the propagating waves is a good indicator of the quality of concrete in various structures. The velocity of concrete is evaluated from the dispersion curves representing the variation of velocities with the propagating waves. In this study, propagating Lamb waves (e.g., A0 Lamb wave mode) were extracted by applying Hanning windows with a size of 2 times of the period at the minima of first dominant time wave signals. The dispersion image was obtained by applying the phase-shift method [30] on the eight windowed time-domain signals measured by the multi-channel IE testing sensor array as follows,

(8) $$S_{w}\left(\omega ,V_{ph},x_{s}\right)=\int e^{-i\left(\frac{\omega }{V_{ph}}\right)x_{r}}[U_{w}\left(r,s,\omega \right)/\left|U_{w}\left(r,s,\omega \right)\right|]dx_{r}$$

where $U_{w}\left(r,s,\omega \right)$ is the Fourier transformation of the time signal $u_{w}^{}\left(r,s,t\right)$; *w* represents windowed signals, r is the location of sensors, s is the location of the impact source, *t* is time, ω is the angular frequency (2πf), and $V_{ph}$ is phase velocity. The integral results in a value in the range of 0 to 1 for a set of frequency and phase velocity. In this study, a dominant propagating wave mode was extracted from the dispersion image of narrow windowed time signals by finding a peak amplitude along the vertical axis for a given frequency as follows,

(9) $$I_{w}^{}\left(\omega ,x_{s}\right)=\underset{V_{ph}}{\arg\max\;}S_{nw}\left(\omega ,V_{ph},x_{s}\right)$$

**Figure 10:**
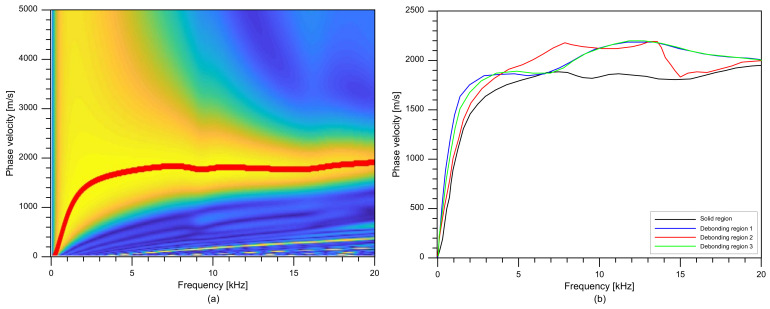
Typical dispersion image and dispersion curves: (**a**) an example of dispersion image measured on the solid concrete region, (**b**) comparison of dispersion curves measured over solid concrete and the three debonding defects.

A resulting dispersion curve, $I_{w}^{}\left(\omega ,x_{s}\right)$, is presented as a red line in Figure 10a, which appears to match with the A0 Lamb mode. Figure 10b compares the dispersion curves extracted from the dispersion images obtained from the solid test region and three concrete regions over debonding defects 1, 2, and 3. It was noticed that the presence of the debonding defects increases the phase velocities in a frequency range of 5 kHz to 20 kHz compared to that of the solid concrete region. 

## 4. Results and Discussion

### 4.1. Velocity of the Propagating Waves in the Track Concrete Layer (TCL) 

**Figure 11:**
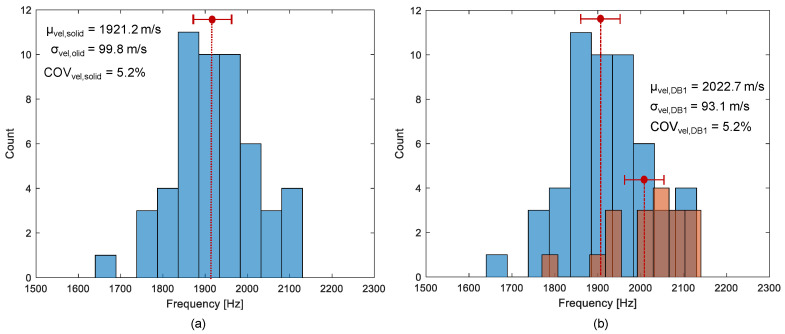
Histograms representing the distribution of the average phase velocity of concrete measured on: (**a**) the solid concrete region (blue), and (**b**) the de-bonded test region 1 (brown).

**Figure 12:**
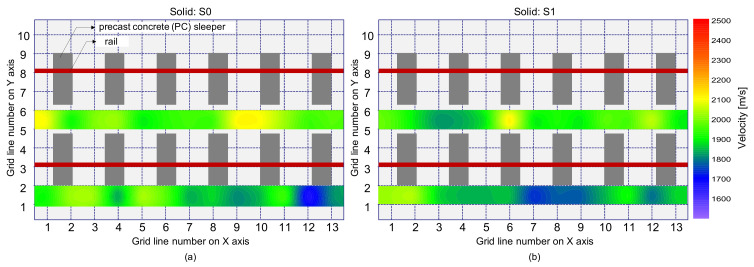
Spatial distribution of the average phase velocity of concrete measured on the solid concrete region: (**a**) using an impact source S0, and (**b**) using an impact source S1.

A histogram presented by blue bars in Figure 11 shows the distribution of average phase velocity measured on the solid concrete region by IE testing using the impact sources located at S0 and S1. The average phase velocity was determined by averaging the phase velocity values in a frequency range of 10 kHz to 20 kHz in a dispersion curve extracted from a dispersion image (see Figure 10a). Each velocity value is associated with a concrete property under the sensor array (i.e., the test region BC in Figure 7b). The mean value and standard deviation of the phase velocity values measured in the solid concrete slab are 1921.0 m/s and 99.8 m/s, respectively, with a coefficient of variation (COV) of 5.2%. About 84% of a total 52 phase velocity values are within 1.5 standard deviation of the mean, which is close to that of the normal distribution (= 85%). Furthermore, the spatial distribution of the average phase velocity is shown in Figure 12, which is dominated by green color, a value within one and a half standard deviations of the mean. The COV of the velocity of concrete is lower than the value measured in the actual concrete slab structures in highway roads (e.g., concrete pavement and concrete bridge decks), which is typically on the order of 10% to 20% [31]. The relatively low COV in this study demonstrates that various experimental uncertainties (fabrication errors, test equipment, and heterogeneity of concrete) were controlled well under a reasonable value. The relatively low COV in this study could be attributed to the fact that the concrete specimen had not been exposed to actual traffic loads, and thus had lower opportunities for damages. Nonetheless, the distribution of velocity in the real-scaled concrete slab track specimen is still useful to better understand the variability of concrete velocity in solid concrete slab tracks with various embedded construction items such as precast concrete sleepers, lattice trusses, reinforcing steels, and various inserts in concrete (see Figure 5b). 

**Figure 13:**
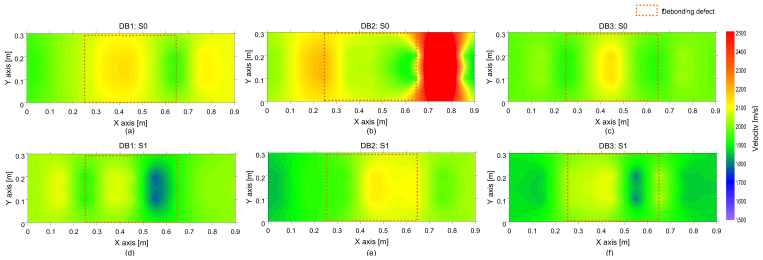
Spatial distribution of the average phase velocity of concrete measured on the de-bonded test regions solid: first (**a**,**d**), second (**b**,**e**) and third (**c**,**f**) columns represent the debonding defects 1, 2 and 3, respectively, and top (**a**–**c**) and bottom (**d**–**f**) rows represent the results measured by impact sources at S0 and S1, respectively.

In contrast, a distribution of the average phase velocity measured over a debonding defect 1 (DB1) is shown as brown bars in Figure 11b. Unlike in the solid test region, the average velocity distribution measured over the de-bonding defect 1 (DB1) is skewed on the left-hand side. The velocity distribution over the de-bonding defects 2 (DB2) and 3 (DB3) were not shown in this article but those showed a similar trend to that ofDB1. Maximum likelihood velocity values measured over the DB1 are in a range of 2050 m/s to 2100 m/s, which is slightly greater than the mean value in solid concrete. The result in this study appears to be opposed to the typical trend of results in the literature that the presence of debonding defects in concrete reduces the velocity of concrete [19]. However, it should be noted that the wavelength of the dominant wave is not large enough, compared to the depth of the debonding defect (~240 mm), to interfere with the top surface of the defect. Therefore, it is reasonable to say that the presence of such a deep debonding defect (~240 mm) at the interface between TCL and HSB could not affect the velocity of concrete. Furthermore, the quality of concrete over a debonding defect in the concrete slab specimen maintained a sound condition. Consequently, the higher value of the concrete velocity can be associated with the interference of the propagating waves with mode converted waves due to the transition of boundary conditions around the debonding defects. Aside from this, it is also observed that the test regions corresponding to higher velocity were not well correlated with the actual location of debonding defects in the velocity map (see Figure 13). Therefore, it can be inferred that a single velocity measurement is not sufficient to accurately identify and characterize a debonding defect at the interface between TCL and HSB. 

### 4.2. Peak Frequency Map

#### 4.2.1. Peak Frequency Based on the Single Sensor Data Analysis

**Figure 14:**
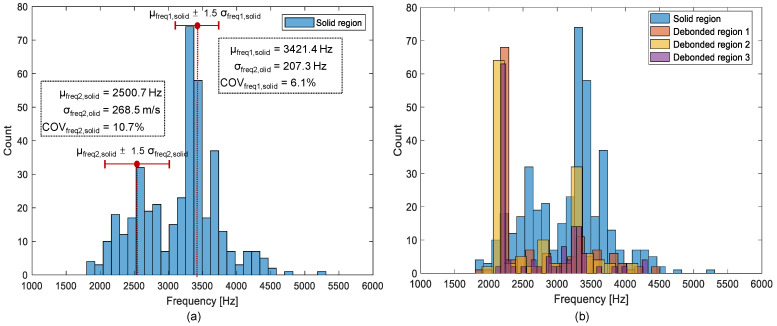
Histograms of peak frequencies obtained from a single accelerometer on: (**a**) the solid region, and (**b**) the debonding regions in the concrete slab track specimen.

**Figure 15:**
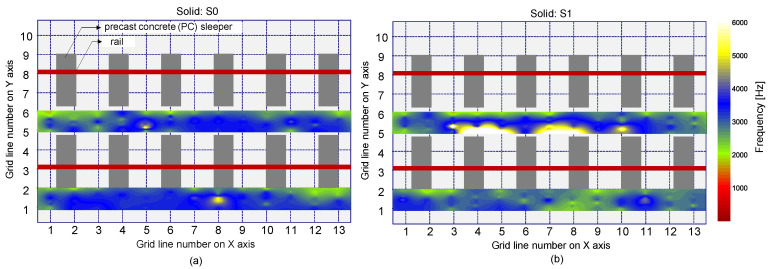
Spatial distribution of peak frequencies obtained from a single accelerometer on the solid region: (**a**) using an impact source at S0, and (**b**) using an impact source at S1.

Figure 14a is a histogram representing the distribution of the peak frequency values measured on the sound concrete region. Each peak frequency value was determined in spectral amplitude data obtained by a single accelerometer. In this study, eight peak frequency values were simultaneously obtained at single testing using the multi-channel IE test device (see Figure 6). A total of 416 peak frequency values were measured on the sound concrete region, which were used to construct the histogram in Figure 14a. There are two distinct peaks in the histogram: the primary peak around 3300 Hz and the secondary peak around 2500 Hz. The presence of two peaks indicates the resulting data come from multiple sources. Provided a Poisson’s ratio of 0.18 for normal concrete and an average concrete P-wave velocity of 3600 m/s, derived from the measured average surface wave velocity of 1921 m/s, the estimated thickness stretch mode frequency of the solid concrete slabs (TCL and HSB layers) with a thickness of 450 mm is determined to be 3140 Hz. Therefore, it can be inferred that the primary frequency around 3300 Hz is corresponding to the thickness of the solid concrete slabs. In the conventional IE test, the test region that is characterized as the full thickness mode is interpreted as a solid concrete region. In this study, the peak frequency data are divided into two groups: the primary group with a frequency value greater than 3000 Hz, and the remaining into the secondary group. The mean value and standard deviation of the peak frequency values associated with the full thickness mode are 3421.4 Hz and 207.3 Hz, respectively, with a COV of 6.1%. About 86.2% of the data within the one-and-a-half standard deviation of the mean value are close to the value of the normal distribution. The spatial distribution of peak frequency values in the solid region is dominated by blue color, which is corresponding to the full thickness mode (see Figure 15). Interestingly, the frequency values greater and lower than the full thickness mode are more frequently observed at the ends of the sensor array. It can be assumed that the secondary peaks are attributed to the presence of an anomaly in concrete such as precast concrete sleeper, reinforcing steel, tie, etc., which is embedded in the TCL. These anomalies could increase the period of resonance mode of non-propagating waves, resulting in a frequency shift in the lower frequency direction. 

**Figure 16:**
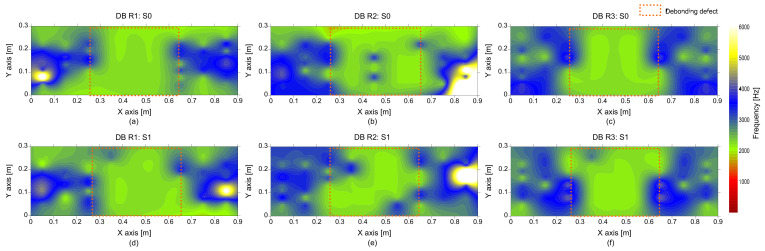
Spatial distribution of peak frequency values obtained from a single accelerometer measured on the de-bonded test regions: first ((**a**,**d**)), second ((**b**,**e**)), and third ((**c**,**f**)) columns represent the debonding defects 1, 2, and 3, respectively, and top ((**a**–**c**)) and bottom ((**d**–**f**)) rows represent the results measured by impact sources at S0 and S1, respectively.

In contrast, histograms representing the distribution of peak frequency values over the three debonding defects (DB1, DB2, and DB3) are shown in Figure 14b. Dominant peak frequency values are concentrated in a narrow frequency range of 2000 Hz to 2200 Hz, with a mean value of 2100 Hz and a standard deviation of 20 Hz (COV = 0.9%). This value is highly compatible with the predicted fundamental frequency of flexural vibration mode by the semi-analytical equation, 2100 Hz, based on the geometry of the debonding defects (*c*/*h* = 1.70, 1.74, and 1.78), and P-wave velocity of 4000 m/s. Therefore, it can be inferred that the dominant peak frequency can be interpreted as the flexural vibration mode of concrete over a debonding defect. Figure 16, representing the spatial variation of peak frequency values, clearly shows the location of the debonding defects at the interface between TCL and HSB which is characterized by green colors, two standard deviations of the mean value. This peak frequency distribution associated with the debonding defects is clearly separated from the primary peak frequency values. However, it is noticed that those overlaps with the lower bound of the secondary peak frequency measured on the sound concrete. Consequently, some sound test regions could be misinterpreted as damaged regions or vice versa. Generally, the error level increases as the overlapped area in the two distributions increases. The presence of the multiple peaks in the solid concrete region could increase difficulties in the interpretation of the IE data in actual concrete slab tracks.

#### 4.2.2. Peak Frequency Based on the Multi-Sensor Data Analysis

**Figure 17:**
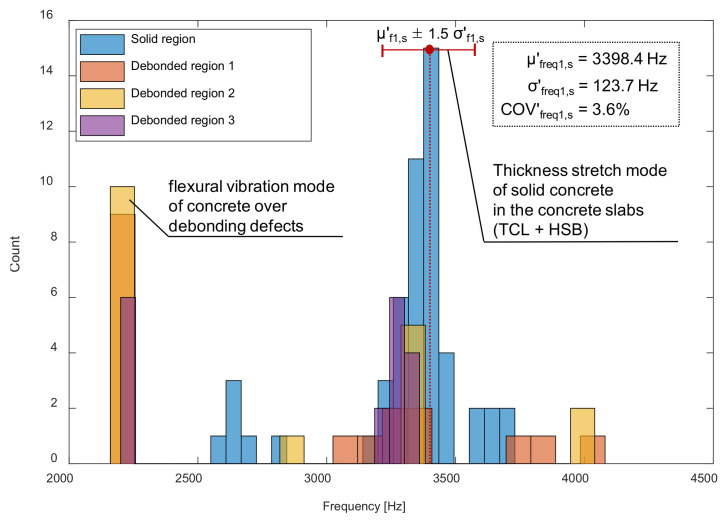
Histograms of peak frequencies obtained from an average of eight accelerometers in the multi-channel IE sensor array on the solid region (blue), the debonding regions 1 (yellow), 2 (orange), and 3 (purple) in the railway concrete slab track specimen.

**Figure 18:**
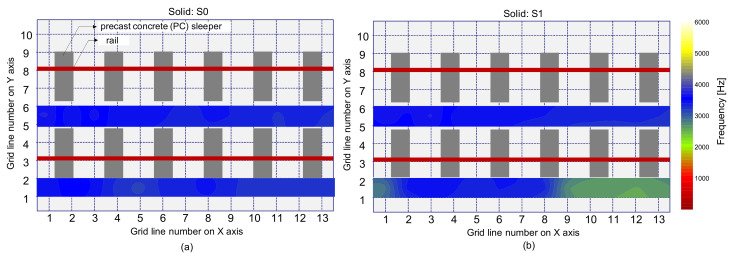
Spatial distribution of the average peak frequency values determined by average of eight spectral amplitude signals measured by the multi-channel IE sensor array over the solid region: (**a**) using an impact source at S0, and (**b**) using an impact source at S1.

**Figure 19:**
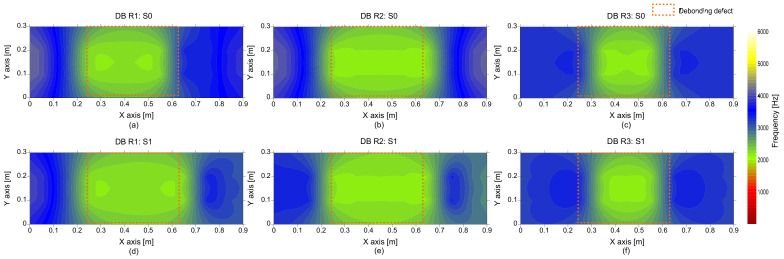
Spatial distribution of the average peak frequency values determined by average of eight spectral amplitude signals measured by the multi-channel IE sensor array over the de-bonded test regions: first (**a**,**d**), second (**b**,**e**), and third (**c**,**f**) columns represent the debonding defects 1, 2, and 3, respectively, and top (**a**–**c**) and bottom (**d**–**f**) rows represent the results measured by impact sources at S0 and S1, respectively.

Figure 17 is a histogram representing the distribution of peak frequency values determined from the average spectral amplitude signal of eight accelerometers (hereafter referred to as the average peak frequency), measured on the solid concrete region. The primary peak frequency is dominated by the average peak frequency distribution. The secondary peak frequency was significantly suppressed in the average peak frequency distribution compared to the peak frequency distribution determined from a single spectral amplitude signal shown in Figure 14a. Theoretically, the noise level of a signal is inversely proportional to the square root of the number of signals that are used in averaging the signals. For an example in this study, the use of eight sensors results in the noise level being suppressed to about 35.3% (1/$\sqrt{8}\sim 0.353)$ of that of the single sensor data. As a result of averaging, the peak frequency map was dominated by blue color, representing one and half standard deviations of the average peak frequency (Figure 18). Furthermore, Figure 19 shows that three debonding defects are clearly identified as green color in the spatial distribution of the average peak frequency values in three test regions including debonding defects. 

### 4.3. Q Factor

**Figure 20:**
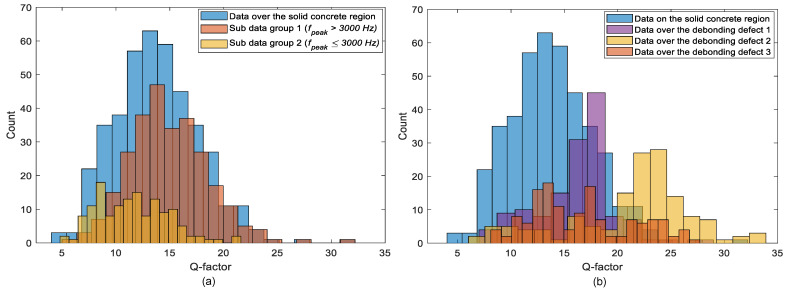
Histograms representing the distribution of *Q* factor values measured using a single accelerometer on: (**a**) the solid concrete region and (**b**) the test region including the debonding defects 1, 2, and 3.

Figure 20 shows histograms that represent the distribution of *Q* factor values determined from spectral amplitude data measured by an individual accelerometer in the multi-channel IE device. *Q* factor distributions obtained in the solid and the three de-bonded regions are presented in Figure 20a,b, respectively. *Q* factor values measured over the solid concrete region, presented as blue bars in Figure 19a, vary in a range of 5 to 20, with a mean value of 13.7 and a standard deviation of 3.9. For a comparison, *Q* factor values over the solid concrete region are sub-divided into two groups (see Figure 20a): one data group associated with the primary peak frequency (*f_peak_* ≥ 3000 Hz), which is shown as brown bars, and the second data group associated with the secondary peak frequency (*f_peak_* < 3000 Hz), which is shown as yellow bars. Furthermore, *Q* factor values obtained over the three debonding defects, DB1, DB2, and DB3, presented as purple, yellow, and brown bars respectively, are shown in Figure 20. It is noticed that the dynamic response of concrete associated with the secondary peak frequency in the solid concrete region tends to result in lower *Q* factor values than those associated with the first peak frequency (full-thickness mode). In addition, dynamic responses over debonding defects result in an even greater *Q* factor compared to those in a full-thickness mode in solid concrete regions. Therefore, it can be inferred that the *Q* factor is a potential parameter that classifies the condition of the concrete in concrete slab tracks. 

**Figure 21:**
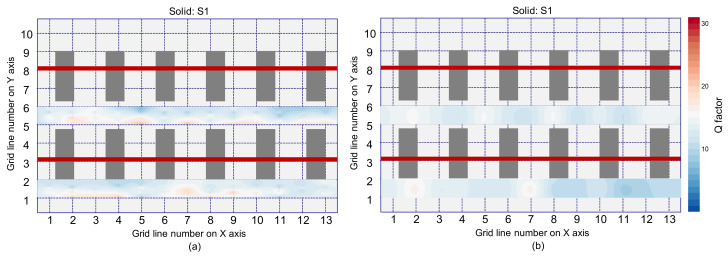
Spatial distribution of *Q* factor values measured over the solid concrete region by an impact source at S1 based on: (**a**) a spectral amplitude measured by a single accelerometer, and (**b**) average of spectral amplitude signals measured by eight accelerometers.

Figure 21 shows the spatial distribution of *Q* factors in the solid test region by IE testing using an impact source located at S1. Figure 21a,b are based on the *Q* factor values measured by a single accelerometer and the averaged *Q* factor values measured by eight accelerometers, respectively. By inspection of the distribution characteristics, the test regions that have *Q* factor values less than 15 are depicted as blue color in the *Q* factor maps, and other test regions are presented as red color. It is observed that most regions in the solid concrete are presented in blue color. Therefore, solid concrete regions can be characterized as dynamic responses with relatively low *Q* factor values (i.e., lower than 15 in this study). 

**Figure 22:**
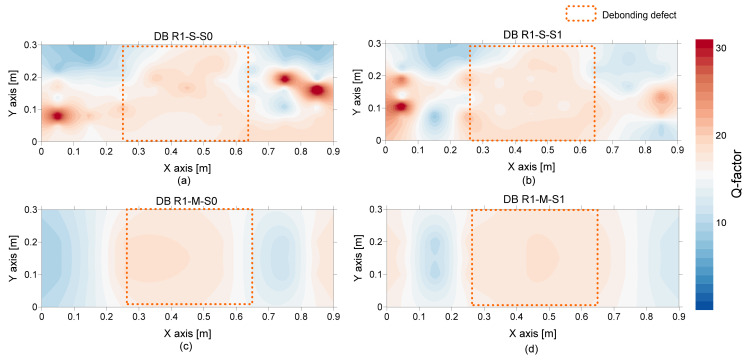
Spatial distribution of *Q*-factor values over the de-bonded test region 1 (DB1): first (**a**,**b**) and second rows (**c**,**d**) show the values based on a single spectral amplitude measured by a single accelerometer and the average spectral amplitude measured by eight accelerometers, respectively, and lest (**a**,**c**) and right (**b**,**d**) columns show the values based on an impact source at S0 and S1, respectively.

On the other hand, the spatial distribution of *Q* factors over the debonding defect 1 are shown in Figure 22. *Q* factor values based on a single accelerometer and multiple accelerometers are shown in the first and second rows, and the values based on an impact-source at S0 and S1 are shown in the first and second columns in Figure 22. With the same color map used in Figure 21, most de-bonded regions are presented in red color. However, some solid test regions are also presented as red color, which can be misinterpreted as de-bonded regions. In this connection, it can be inferred that the *Q* factor alone is not sufficient to classify the solid and de-bonded test regions in concrete specimens. However, it is worthy to note that the test regions classified as unknown areas in the frequency maps are presented as solid regions in the *Q* factor maps. Therefore, it can be inferred that the *Q* factor values are a potential parameter that could improve the accuracy of condition assessment when those values are supplementarily used with peak frequency values. 

### 4.4. Evaluation of Parameters

This section evaluates the effectiveness of individual IE parameters for classifying the debonding defects and solid concrete in the concrete slab track specimen. A bilinear classification model was used to evaluate various characteristic parameters in this study: the five individual IE parameters ($\;V_{R}$, $f_{peak}$,$\;{\bar{f}}_{peak}$, Q, and $\bar{Q}$), and two combined parameters ($f_{peak}+Q$, and ${\bar{f}}_{peak}$+$\;\bar{Q}$). A bilinear model classifies the test region into solid (negative) or de-bonded (positive) regions by comparing a parameter value with a threshold value. Table 1 and Table 2 summarize the evaluation criteria for the bilinear prediction models based on single and dual IE testing parameters, respectively. Consequently, test regions are divided into four groups: true positive (TP), false negative (FN), true negative (TN), and false positive (FP). TP and TN represent results where the model correctly predicts the positive (de-bonded) and negative (solid) classes, respectively. Similarly, FN and FP represent results where the model incorrectly predicts the negative and positive classes, respectively.

**Figure 23:**
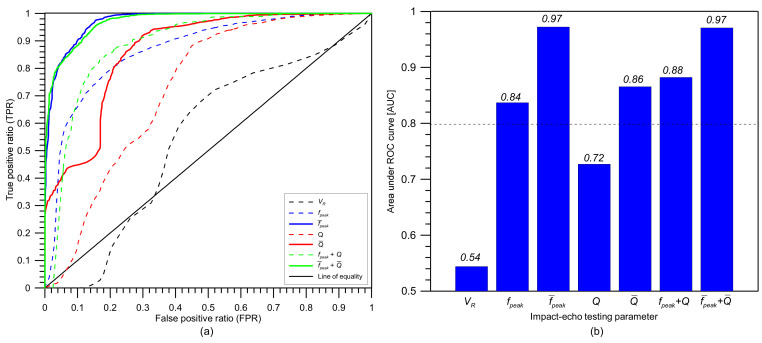
Comparison of performance of bilinear classification models based on various IE parameters from multi-channel IE testing in this study: (**a**) receiver operating characteristic (ROC) curves, and (**b**) Area under ROC curve (AUC).

Figure 23 a represents the receiver operating characteristic (ROC) curves for the bilinear classification models with various characteristic parameters from multi-channel IE testing in this study. An ROC curve represents the relationship between true positive ratio (TPR = TP/(TP + FN)) with false positive ratio (FPR = FP/(FP + TN)) determined from various threshold values of a parameter. Figure 23b shows the area under the ROC curves (AUC) associated with different parameters. The AUC value is a good indicator of the overall performance of the classification model in a certain range of threshold values compared to a perfect model, with an AUC value of 1.0. The parameter that results in the AUC value closer to 1.0 is interpreted as to be a more effective parameter in a classification model. Overall, the prediction models based on the averaged values from multi-channel data exhibit higher AUC values than those based on single-channel data. To be more specific, the equally highest AUC values (0.97) resulted from the prediction model based on the average peak frequency (${\bar{f}}_{peak}$) and the combined average peak frequency and averaged *Q* factor (${\bar{f}}_{peak}$ and $\bar{Q}$). The effectiveness of using the *Q* factor as a supplementary parameter is more clearly observed in the prediction model based on single-channel IE data. The use of dual parameters ($f_{peak}$ and Q) improves the AUC value by about 5% (or 18%) compared to the model based on a single parameter of $f_{peak}$ (or Q). 

The results in this study are obtained from the debonding defects with a limited range of *c/h* values (*c*/*h* = 1.70, 1.74, and 1.78). Debonding defects with larger areal extensions, with greater *c/h* value, would result in lower peak frequency. Consequently, the performance of the prediction model based on peak frequency values ($f_{peak}$ or ${\bar{f}}_{peak}$) would be valid for identifying the debonding defects with larger areal sizes (i.e., larger *c*/*h*). However, it should be noted that the overlapped area between the secondary peak frequencies in the solid region (2300 Hz to 3000 Hz) and peak frequency associated with flexural oscillation mode becomes greater as the areal size of debonding defects decreases. Therefore, it can be inferred that the performance of the prediction model that is only based on peak frequency values could be reduced when it is applied to detecting debonding defects with smaller areal dimensions considered in this study. In this case, it can be observed that the use of a dual parameter model would be helpful to classify the flexural vibration mode and the secondary peak frequency in solid concrete regions (2300 Hz to 3000 Hz). Considering the characteristic frequency of sound concrete slabs is about 3420 Hz (μ_freq1,solid_) and standard deviation about 207 Hz (σ_freq1,solid_), it can be reasonably said that the width of debonding defects should be sufficiently small to result in the flexural vibration mode frequency lower than 3000 Hz (~μ_freq1,solid_ − 1.5 σ_freq1,solid_). From Figure 4, it can be found that the normalized width of a debonding defect (*c*/*h*) should be greater than 1.0 to satisfy the criterion. Consequently, the minimum width of debonding defects that can be detected by the proposed method is about 240 mm (= the thickness of TCL). 

## 5. Conclusions

In this study, a multi-channel IE test was applied to the real size railway concrete slab specimen that was fabricated with the same design and materials of concrete slab tracks in the Korea high-speed railway system. Multi-channel signal data were reduced to several critical parameters (i.e., the velocity of concrete, peak frequency, and damping properties) that describe the oscillatory behavior of solid concrete and concrete over debonding defects. The distribution of the multiple IE parameters was obtained from the solid and de-bonded regions. The effectiveness of multiple IE parameters is compared by evaluating the performance of a bilinear prediction model with various IE parameters obtained from the multi-channel IE test in this study. The specific descriptions of the primary results in this study are drawn as follows,

(1) It was observed that statistical results associated with the distribution of velocity could differentiate the solid regions and test regions with debonding defects. Therefore, the velocity measurement can be useful to evaluate the overall condition of the test slabs. However, special care is needed to identify the location of the debonding defect at the interface between TCL and HSB based on the single velocity value.
(2) Overall, the peak frequency map based on the single-channel IE testing is effective for differentiating solid concrete and de-bonded regions in the concrete slab track specimen. However, the presence of multiple peaks in the solid concrete region could increase difficulties in the interpretation of the IE data in actual concrete slab tracks. The primary peak (around 3300 Hz) is corresponding to the full thickness mode, which is usually interpreted as the characteristic frequency of the solid region in IE testing. The secondary peak frequency group (around 2000 Hz to 3000 Hz) is not expected by the IE theory. Dynamic response of concrete over debonding defects is characterized by flexural vibration mode with a peak frequency range of 2100 Hz to 2200 Hz. It was observed that the distribution of flexural vibration mode frequency is overlapped with the lower portion of secondary peak frequency in solid concrete, which could increase the level of error in the prediction model based on peak frequency values based on the single-channel IE testing.
(3) It was demonstrated that the use of average peak frequency measured by multiple IE testing devices is effective to suppress the unknown peak frequency in the solid concrete. As a result of averaging, the peak frequency map was generally represented by blue color, representing one and half standard deviations of the average peak frequency. Three debonding defects are clearly identified as green color in the spatial distribution of the average peak frequency values in the three test regions, including debonding defects.
(4) *Q* factor is a potential parameter that can separate the flexural oscillation mode and the secondary peak frequency in the solid concrete when difficult to accurately classify based on differences in peak frequency values. It is noticed that the dynamic response of concrete associated with the secondary peak frequency in the solid concrete region tends to result in lower *Q* factor values than those associated with the first peak frequency (full-thickness mode). Furthermore, dynamic responses over debonding defects result in even greater *Q* factor values compared to those in full-thickness mode at solid concrete regions.
(5) Overall, the prediction models based on the averaged values from multi-channel data exhibit higher AUC values than those based on single-channel data. Specifically, the equally highest AUC values (0.97) are resulted from the prediction model based on the average peak frequency (${\bar{f}}_{peak}$) and the combined average peak frequency and averaged *Q* factor (${\bar{f}}_{peak}$ and $\bar{Q}$). The effectiveness of using the *Q* factor as a supplementary parameter is more clearly observed in the prediction model based on single-channel IE data. The use of dual parameters ($f_{peak}$ and Q) improves the AUC value by about 5% (or 18%) compared to the model based on a single parameter of $f_{peak}$ (or Q).

## Figures and Tables

**Table 1:** Summary of bilinear models based on single IE testing parameters.

Prediction	Peak Frequency	Velocity or *Q* Factor
1 (de-bonded region)	*f_peak_* < *τ_freq_*	*V_R_* (or *Q*) ≥ *τ_Vel_* (or *τ_Q_*)
0 (solid region)	*f_peak_* ≥ *τ_freq_*	*V_R_* (or *Q*) < *τ_Vel_* (or *τ_Q_*)

Note: *τ* is a threshold value, the subscripts *freq*, *vel*, and *Q* represent peak frequency, velocity, and *Q* factor, respectively.

**Table 2:** Summary of bilinear models based on dual IE testing parameters (combined *f_peak_* and *Q* factor).

Prediction	Combination of Peak Frequency and *Q* Factor Values
1 (de-bonded region)	*f_peak_* < *τ_freq_* and *Q* > *τ_Q_*
0 (solid region)	*f_peak_* ≥ *τ_freq_*

## Data Availability

Data are contained within the article. But the data presented in this study are also available on request from the corresponding author.

## References

[1] Gautier P.-E. (2015). Slab track: Review of existing systems and optimization potentials including very high speed. Constr. Build. Mater..

[2] Zhu S., Wang M., Zhai W., Cai C., Zhao C., Zeng D., Zhang J. (2018). Mechanical property and damage evolution of concrete interface of ballastless track in high-speed railway: Experiment and simulation. Constr. Build. Mater..

[3] Vu L., Jang D.D., Kang Y.-S. (2021). Assessment of structural dynamic response and vehicle-track interaction of precast slab track systems. Appl. Sci..

[4] Zhong Y., Gao L., Zhang Y. (2018). Effect of daily changing temperature on the curling behavior and interface stress of slab track in construction stage. Constr. Build. Mater..

[5] Zhu S., Cai C. (2014). Interface damage and its effect on vibrations of slab track under temperature and vehicle dynamic loads. Int. J. Non-Linear Mech..

[6] Ren J., Yang R., Wang P., Yong P., Wen C. (2014). Slab Upwarping of twin-block slab track on subgrade-bridge transition section. J. Transp. Res. Board.

[7] Korea Rail Network Authority (2016). Concrete Railway Structures.

[8] Korea National Reailway KR Technology: Track System of High-Speed Railway. https://english.kr.or.kr/sub/info.do?m=0303.

[9] Kang K.-D., Kang G.H., Lee B.S., Yoo H.S., Jung Y.W. (2014). Korea’s High-Speed Rail Construction and Technology Advances.

[10] Chun H.-K. (2016). Study of the Behavior of Concrete Slab Track of High Speed Train on Earthwork According to the Variation of Train Speed and Axle Load. Graduate School of Railway.

[11] Freudenstein S. (2010). RHEDA 2000^®^: Ballastless track systems for high-speed rail applications. Int. J. Pavement Eng..

[12] Bachmann H., Mohr W., Kowalski M. (2003). The RHEDA 2000 ballastless track system. Eur. Railway Rev..

[13] Xu Y., Yan D., Zhu W., Zhou Y. (2020). Study on the mechanical performance and interface damage of CRTS II slab track with debonding repairment. Constr. Build. Mater..

[14] Su C., Liu D., Ding C., Gong C., Zhao P., Liu X. (2018). Experimental study on bond. Performances of track slab and mortar based on DIC technology. KSCE J. Civil Eng..

[15] Jang S.Y., Choi S.S. Crack and Damage Patterns of Concrete Track of Kyeong-Bu High-Speed Railways (KSR2014S278). Proceedings of the 2014 Autumn Conference and Annual Meeting of the Korean Society of Railway.

[16] ASTM (2018). Standard Practice for Measuring Delaminations in Concrete Bridge.

[17] Oh T., Popovics J.S., Sim S.-H. (2013). Analysis of vibration for regions above rectangular delamination defects in solids. J. Sound Vib..

[18] Oh T.K. (2012). Defect Characterization in Concrete Elements Using Vibration Analysis and Imaging. Civil, Environmental, and Architectural Engineering.

[19] Kee S.-H., Lee J.-W., Candelaria M.D. (2020). Evaluation of delamination in concrete by IE testing using multi-channel elastic wave data. Sensors.

[20] Kee S.-H., Gucunski N. (2016). Interpretation of flexural vibration modes from impact-echo testing. J. Infrastruct. Syst..

[21] Oh T.K., Kee S.H., Arndt R.W., Popovics J.S., Zhu J. (2013). Comparison of NDT methods for assessment of a concrete bridge deck. J. Eng. Mech. ASC.

[22] Gucunski N., National Research Council (2013). Nondestructive Testig to Identify Concrete Bridge Deck Deterioration.

[23] Kee S.-H., Oh T., Popovics J.S., Arndt R.W., Zhu J. (2012). Nondestructive bridge deck testing with air-coupled impact-echo and infrared thermography. J. Bridge Eng. ASCE.

[24] Sansalone M., Carino N.J. (1989). Detecting delaminations in concrete slabs with and without overlays using the impact-echo method. ACI Mater. J..

[25] Sansalone M. (1997). Impact echo: The complete story. ACI Struct. J..

[26] Sansalone M.J., Strett W.B. (1997). Impact-Echo: Nondestructive Evaluation of Concrete and Masonry.

[27] Schubert F., Köhler B. (2008). Ten lectures on impact-echo. J. Nondestruct. Eval..

[28] Gibson A., Popovics J.S. (2005). Lamb wave basis for impact-echo method analysis. J. Eng. Mech. ASCE.

[29] Hayashi T., Kawashima K. (2002). Multiple reflections of Lamb waves at a delamination. Ultrasonics.

[30] Park C.B., Miller R.D., Xia J. (1998). Imaging Dispersion Curves of Surface Waves on Multichannel Record. SEG Technical Program Expanded Abstracts.

[31] Gucunski N., Kee S., La H., Basily B., Maher A. (2015). Delamination and concrete quality assessment of concrete bridge decks using a fully autonomous RABIT paltform. Struct. Monit. Maint..

